# The Beat

**DOI:** 10.1289/ehp.121-a77

**Published:** 2013-03-01

**Authors:** Erin E. Dooley

**Affiliations:** Erin E. Dooley, MA, is a staff writer for *EHP*.

## Interagency Report Urges a Focus on Breast Cancer Prevention

In February 2013 the Interagency Breast Cancer and Environmental Research Coordinating Committee released recommendations for investigating and mitigating environmental causes of breast cancer.^1^ The committee’s report highlights the need for more emphasis on prevention—not just diagnosis and treatment—and suggests that a national breast cancer prevention strategy be developed that would increase and better coordinate the federal government’s investments in this area. The American Cancer Society estimated nearly 227,000 new cases of breast cancer would be diagnosed and nearly 40,000 women would die of the disease in the United States in 2012.^2^

**Figure:**
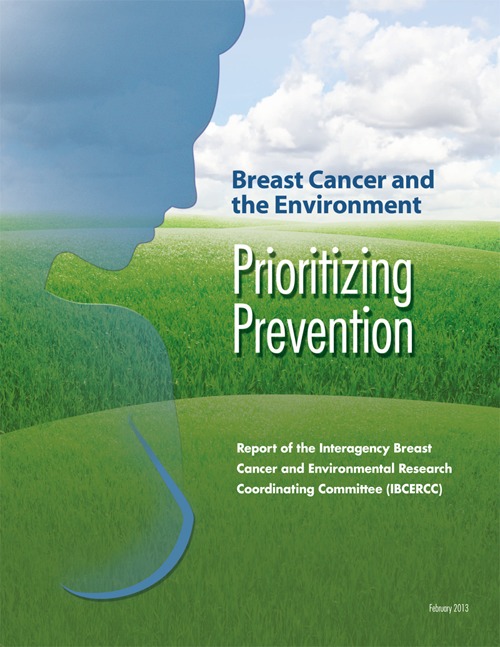
BCERCC

## Warm Winters Could Influence  Flu Seasons

An analysis of climate trends and U.S. influenza seasons dating back to 1997–1998 has found that unusually warm winters tended to be followed by flu seasons marked by their severity and early onset.^3^ The authors of the study suggest that fewer people getting the flu during warm winters may lead to an unusually large number of susceptible people in the next flu season. This could drive earlier and more severe epidemics—a situation exacerbated if outbreaks occur before most people have a chance to get their yearly vaccination.

## Ranking Sources of Foodborne Illness

More than 9 million U.S. cases of foodborne illness are caused by pathogens each year.^4^ A CDC analysis of such illnesses for the period 1998–2008 estimates that more deaths (19%) were associated with poultry than with any other food type and that 46% of illnesses were linked with produce.^5^ Of the latter, 22% were linked to leafy greens. The analysis also identified norovirus as causing the most outbreaks and the most cases of illness. These findings provide a starting point to help the food industry and government agencies prioritize efforts to reduce foodborne illness.

**Figure:**
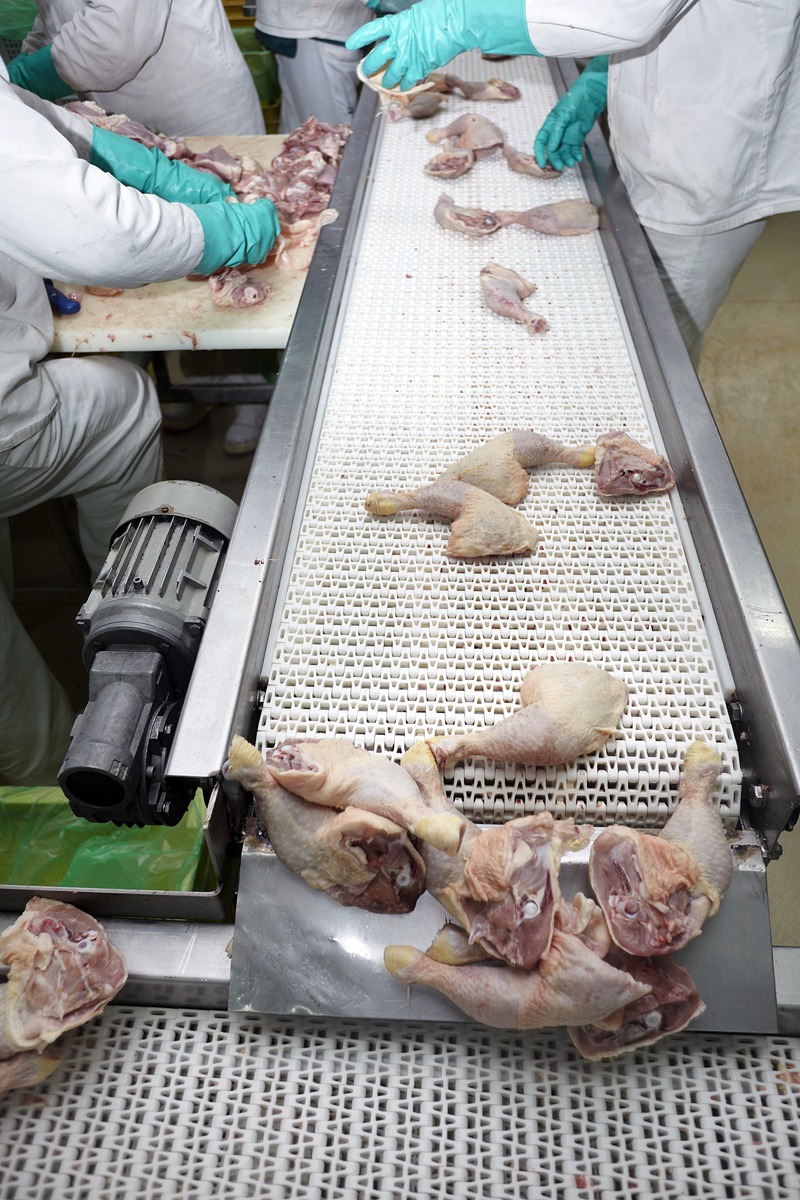
New data may help companies target efforts to prevent foodborne illnesses. © Shutterstock

## California Revisits Flame Retardant Regs

In response to concerns about potential health effects of flame retardants, California officials have proposed updated regulations for these chemicals in upholstered furniture.^6^ Under the proposed regulations, foam cushions and other furniture components would need to withstand a smoldering cigarette rather than the open flame mandated by current law. (State officials say this is a more realistic scenario for home fires.) The proposed rules would therefore reduce the amount of flame retardants needed in furniture. To further curtail children’s exposure, 17 baby and infant products would be exempt from having to meet the new flammability standards.

**Figure:**
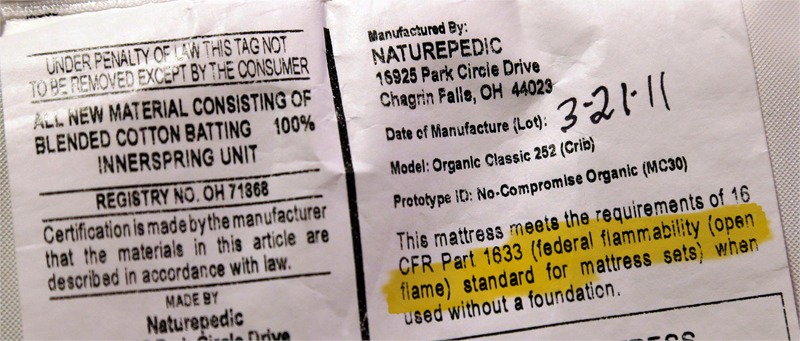
New data may help companies target efforts to prevent foodborne illnesses. © Elaine Thompson/AP/Corbis

## Ozone Exposure and Risk of Preeclampsia

Swedish researchers have estimated that 1 in every 20 cases of preeclampsia may be linked to exposure to ground-level ozone during the first trimester of pregnancy.^7^ The study of 121,000 singleton births found a slightly greater association in mothers who had asthma. Preeclampsia affects women later in pregnancy and involves elevated blood pressure and protein in the urine. It is more prevalent in developing countries and, with other hypertensive disorders, has been identified as a leading cause of perinatal deaths among children in these countries.^8^
